# Association of *CD247 (CD3ζ)* gene polymorphisms with T1D and AITD in the population of northern Sweden

**DOI:** 10.1186/s12881-016-0333-z

**Published:** 2016-10-04

**Authors:** Dan Holmberg, Karin Ruikka, Petter Lindgren, Mats Eliasson, Sofia Mayans

**Affiliations:** 1 Department of Medical Biosciences - Medical Genetics, Umeå University, SE-901 85 Umeå, Sweden; 2 EMV, Immunology, BMC, Lund University, SE-221 00 Lund, Sweden; 3 Department of Medicine, Sunderby Hospital, SE-971 80 Luleå, Sweden; 4 Department of Public Health and Clinical Medicine, Umeå University, SE-901 85 Umeå, Sweden; 5 Department of Clinical Microbiology, Division of Immunology, Umeå University, Building 6C, SE-90185 Umeå, Sweden

**Keywords:** Linkage, Association, Family, Type 1 diabetes, Autoimmune thyroid disease

## Abstract

**Background:**

T1D and AITD are autoimmune disorders commonly occurring in the same family and even in the same individual. The genetic contribution to these disorders is complex making uncovering of susceptibility genes very challenging. The general aim of this study was to identify loci and genes contributing to T1D/AITD susceptibility. Our strategy was to perform linkage and association studies in the relatively genetically homogenous population of northern Sweden. We performed a GWLS to find genomic regions linked to T1D/AITD in families from northern Sweden and we performed an association study in the families to test for association between T1D/AITD and variants in previously published candidate genes as well as a novel candidate gene, *CD247*.

**Methods:**

DNA prepared from 459 individuals was used to perform a linkage and an association study. The ABI PRISM Linkage Mapping Set v2.5MD10 was employed for an initial 10-cM GWLS, and additional markers were added for fine mapping. Merlin was used for linkage calculations. For the association analysis, a GoldenGate Custom Panel from Illumina containing 79 SNPs of interest was used and FBAT was used for association calculations.

**Results:**

Our study revealed linkage to two previously identified chromosomal regions, 4q25 and 6p22, as well as to a novel chromosomal region, 1q23. The association study replicated association to *PTPN22*, *HLA-DRB1*, *INS*, *IFIH1*, *CTLA4* and *C12orf30*. Evidence in favor of association was also found for SNPs in the novel susceptibility gene *CD247*.

**Conclusions:**

Several risk loci for T1D/AITD identified in published association studies were replicated in a family material, of modest size, from northern Sweden. This provides evidence that these loci confer disease susceptibility in this population and emphasizes that small to intermediate sized family studies in this population can be used in a cost-effective manner for the search of genes involved in complex diseases. The linkage study revealed a chromosomal region in which a novel T1D/AITD susceptibility gene, *CD247,* is located*.* The association study showed association between T1D/AITD and several variants in this gene. These results suggests that common susceptibility genes act in concert with variants of *CD247* to generate genetic risk for T1D/AITD in this population.

**Electronic supplementary material:**

The online version of this article (doi:10.1186/s12881-016-0333-z) contains supplementary material, which is available to authorized users.

## Background

Type 1 diabetes mellitus (T1D) and autoimmune thyroid disease (AITD) are autoimmune disorders under complex genetic control [1]. There is a well-known association between T1D and AITD, and both diseases often occur in the same family and even in the same individual, suggesting common genetic causes [1–4]. Several genome-wide linkage scans (GWLS) have been performed to search for genes contributing to T1D [5–10], but to date, no GWLS has been performed on the population of northern Sweden. An internal expansion of the northern Swedish population, together with a low frequency of immigration and a high frequency of consanguineous marriages has resulted in a relatively genetically homogeneous population [11–13]. This genetic composition coupled with other factors, such as the Swedish healthcare system and detailed church records, have enabled the successful use of this population in genetic studies of complex diseases, such as T1D [14, 15], type 2 diabetes [16, 17] and stroke [18]. Genome-wide association (GWA) studies have revealed several risk factor genes/loci for T1D, AITD and a number of other diseases [19–25]. These studies have found convincing evidence of associations of several loci with T1D: *HLA class II* genes on chromosome 6p21, *INS* on 11p15, *CTLA4* on 2q33, *PTPN22* on 1p13, the *IL2Rα* region on 10p15 and the *IFIH1* region on 2q24. Multiple novel chromosomal regions, including 12q13, 12q24, 16p13 and 18p11, have been associated with T1D in both family-based studies and case-control cohorts [19, 21, 23, 26]. Several of the identified risk genes are immunoregulatory in nature, and many of these genes and/or associated biological pathways have also been observed to overlap with findings from animal models of T1D, notably the non-obese diabetic (NOD) mouse model.

Both T1D and AITD develop through a process mediated by T lymphocytes [1] and signaling through the T cell receptor (TCR)/CD3 complex is required for the activation of T cells. The CD3 complex consists of two CD3ε, one CD3γ, one CD3δ and two CD3ζ subunits. Phosphorylation of immunoreceptor tyrosine-based activation motifs (ITAMs) in the CD3 complex is one of the earliest detectable events occurring after TCR engagement [27, 28]. Several of the genes associated to T1D and/or AITD, such as *PTPN22*, *CTLA4* and *IL2/IL2RA* are important for T cell activation, regulation and function [29–32].

In this study, linkage and association analyses of multiplex families from northern Sweden have been employed to find genetic regions and variants contributing to T1D/AITD in the families. We report linkage to T1D/AITD for three chromosomal region and we replicate the previously reported association for T1D/AITD with single nucleotide polymorphisms (SNPs) in *PTPN22*, *HLA-DRB1*, *INS*, *IFIH1*, *CTLA4* and *C12orf30* [21, 23, 24, 33, 34]. We also report association to T1D/AITD for SNPs in the novel susceptibility gene *CD247*.

## Methods

### Families

The initial 10-centimorgan (cM) GWLS included a total of 42 families with 184 family members (54 patients with T1D, 48 patients with AITD, 12 patients with both disorders and 70 unaffected family members) from northern Sweden. Population-based registers, the autoimmune disease register at Sunderby Hospital and the Swedish childhood diabetes registry [35] were used to identify T1D or AITD probands below the age of 50. First-degree relatives with T1D or AITD were then identified, and diabetes diagnoses were confirmed by examining the medical records of both probands and relatives. Genotyping of regions with an initial logarithm of odds (LOD) score higher than 1.5 was further performed in 275 individuals (69 patients with T1D, 62 patients with AITD, 8 patients with both disorders 136 unaffected family members) from an additional 55 families. In a family-based association study, all 459 individuals from the 97 families were genotyped and analyzed. This study was conducted with the approval of the regional ethical review board, and informed consent was obtained from all participants. Clinical characteristics of the study material can be found in Additional file 1: Tables S1 and S2.

### DNA extraction

Genomic DNA was prepared from buffy coat samples using the FlexiGene DNA Kit (Qiagen, Venlo, the Netherlands). DNA extraction was performed according to the manufacturer’s instructions.

### Genome-wide scan

An ABI linkage panel set with an average spacing of 10 cM (ABI PRISM Linkage Mapping Set v2.5MD10, Applied Biosystems, Foster City, CA, USA) was used for the initial 10-cM GWLS. PCR products were analyzed on an ABI PRISM 3730 DNA Sequencer, and genotypes were analyzed using GeneMapper v3.7 (Applied Biosystems, Foster City, CA, USA). The largest gap in the initial GWLS was 14.7 cM. Additional markers from the ABI PRISM Linkage Mapping 5-cM Set, and markers ordered from DNA Technology (Aarhus, Denmark) were typed on chromosomes 1, 4 and 6 in the regions with an initial LOD score higher than 1.5.

### SNP genotyping

A GoldenGate Custom Panel containing 79 SNPs of interest (Additional file 1: Table S3) was purchased from Illumina (San Diego, CA), and genotyping was performed according to the manufacturer’s instructions. SNPs were analyzed using Illumina Beadstation 500GX, and genotypes were analyzed using BeadStudio v.3 (Illumina, San Diego, CA). For each SNP, 8.5 % of the total samples were run in duplicate, and 100 % concordance was observed between duplicates. A genotyping success rate ranging from 79 % to 99 % was obtained for the SNPs under investigation (Additional file 1: Table S3).

### Statistics

For the GWLS, model-free multipoint linkage analysis was performed using Merlin, and allele frequencies were estimated from all genotyped individuals [36]. Three models were used for the statistical calculations. In disease model 1, individuals with T1D were set as affected. In disease model 2, individuals with T1D, individuals with AITD and individuals with both diseases were set as affected. In disease model 3, individuals with AITD were set as affected. Association analysis in the family-based material was performed using FBAT v2.0.4Q (http://www.hsph.harvard.edu/fbat/default.html), which is a software package used for computing Family Based tests of Association. *P*-values were not corrected for multiple comparisons. A SNP with a *p*-value <0.05 was considered associated to T1D/AITD. The distribution of alleles in the SNPs under investigation did not deviate significantly from Hardy-Weinberg equilibrium in the control group.

## Results

**Fig. 1:**
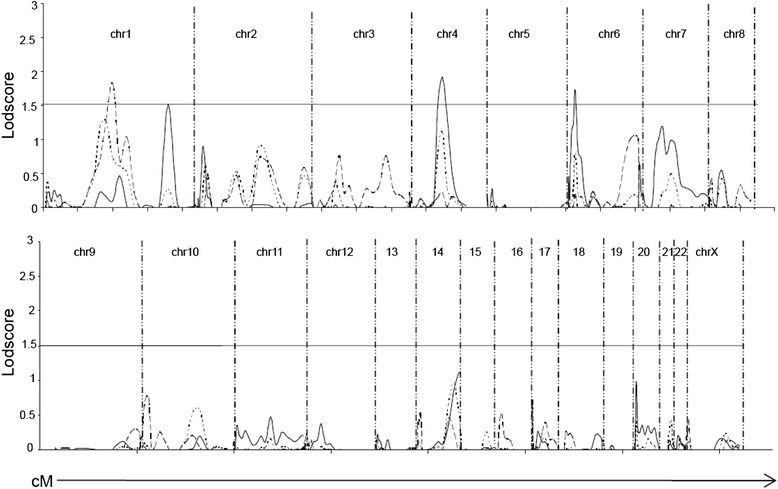
Genome-wide multipoint allele-sharing LOD scores. The initial 10-cM GWLS was performed in 184 individuals from 42 families. The vertical axis denotes the LOD scores, and the horizontal axis denotes the relative cM position on each chromosome (chr) (Genethon Map). Solid line, disease model 1; dotted line, disease model 2; dashed line, disease model 3

**Fig. 2:**
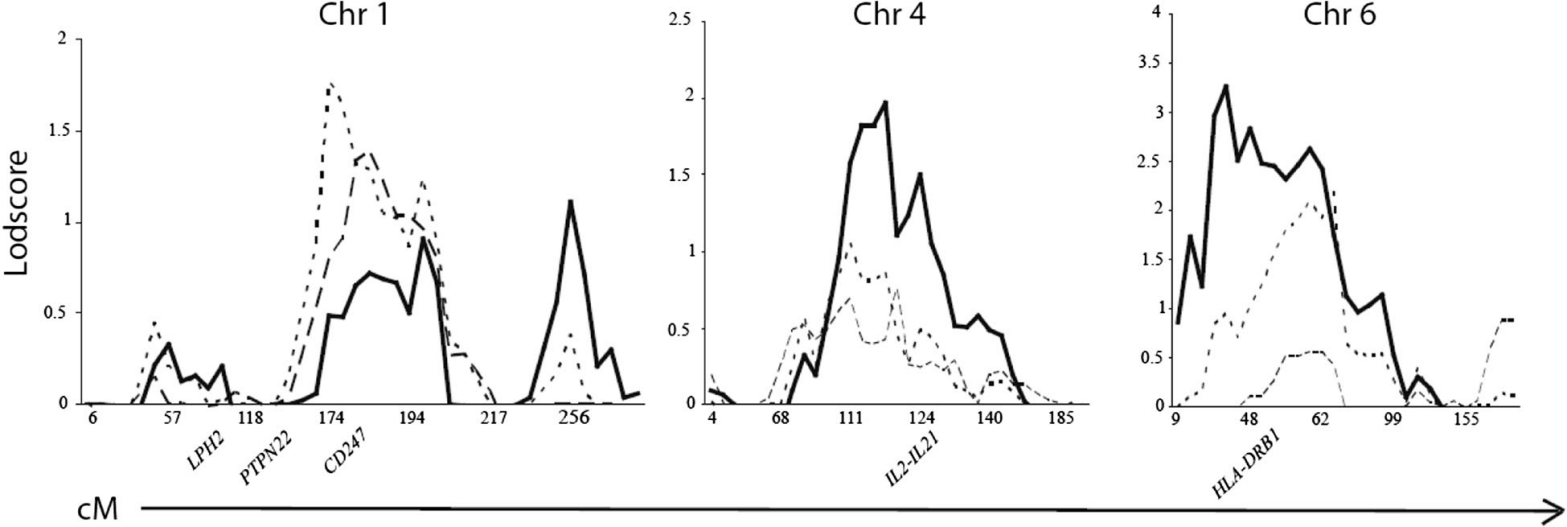
Multipoint allele-sharing LOD scores after fine mapping. Multipoint allele-sharing LOD scores after fine mapping of regions with an LOD score > 1.5 in the initial 10-cM GWLS and with the addition of 275 new individuals. The vertical axis denotes LOD scores, and the horizontal axis denotes relative cM position (Genethon map). Genetic location of genes included in the association study is shown on the horizontal axis. Solid line, disease model 1; dotted line, disease model 2; dashed line, disease model 3

In the first family recruitment, 42 families were identified, and 184 individual samples (54 patients with T1D, 48 patients with AITD, 12 patients with both disorders and 70 unaffected family members) were collected to be included in a 10-cM GWLS. The initial linkage analysis revealed allele-sharing LOD scores > 1.5 (arbitrary chosen cut-off) at three locations: 1q23 (LOD = 1.99 at marker D1S452), 4q26 (LOD = 1.92 at marker D4S402) and 6p21 (LOD = 1.78 at marker D6S1549) (Fig. 1). Fine mapping of the three regions of interest and the addition of 275 individuals (69 patients with T1D, 62 patients with AITD, 8 patients with both disorders and 136 unaffected family members) from 55 new families increased the LOD score at 4q25 (LOD = 1.97 at marker D4S1616) and 6p22 (LOD = 3.26 at marker D6S422) and decreased the LOD score slightly at 1q23 (LOD = 1.77 at marker D1S484) (Fig. 2).

SNPs chosen for the association study were selected based on the results from our GWLS in combination with SNPs associated to T1D/AITD in previously published GWA studies [19–23]. 79 SNPs, 42 previously published associated SNPs and 37 SNPs within the novel susceptibility gene, *CD247*, were selected to be included on an Illumina GoldenGate Custom Panel (Additional file 1: Table S3). DNA from 459 individuals, including 123 patients with T1D, 110 patients with AITD, 20 patients with both disorders and 206 healthy individuals from 97 families, were genotyped using this panel. SNPs were tested for deviation from Hardy-Weinberg equilibrium in the control group using Haploview 3.2 [37]. Of the 79 SNPs included in the panel, 13 were associated (nominal *p* < 0.05) with T1D, while 6 were associated with AITD (nominal *p* < 0.05) (Table 1). Evidence in favor of association with T1D was obtained for SNPs in *PTPN22* (rs6679677 p = 4.5 × 10^-4^, rs2476601 p = 4.5 × 10^-4^)*, CD247* (rs6668182 *p* = 0.007, rs2988276 *p* = 3.8 × 10^-5^, rs7523351 *p* = 0.012, rs10918695 *p* = 0.006, rs12144621 *p* = 0.036, rs863455 *p* = 0.025, rs704852 *p* = 0.029), *HLA-DRB1* (rs9270986 p = 1.8x10^-6^) and *INS* (rs1004446 *p* = 0.011, rs6356 *p* = 0.020, rs7111341 *p* = 0.021). Evidence in favor of association with AITD was obtained for SNPs in *CD247* (rs17534481 *p* = 0.018, rs12095738 *p* = 0.022), *IFIH1* (rs1990760 *p* = 0.032), *CTLA4* (rs3087243 *p* = 0.039), *INS* (rs6356 *p* = 0.014) and *C12orf30* (rs17696736 *p* = 0.032). *P*-values were not corrected for multiple testing.

**Table 1:** SNPs showing association to TID or AITD in the family material from northern Sweden

Disease	Chr	SNP	Gene	*p*-value
T1D	1p13	rs6679677	PTPN22	4.5x10^-4^
T1D	1p13	rs2476601	PTPN22	4.5x10^-4^
T1D	1q24	rs6668182	CD247	0.007
T1D	1q24	rs2988276	CD247	3.8x10^-5^
T1D	1q24	rs7523351	CD247	0.012
T1D	1q24	rs10918695	CD247	0.006
T1D	1q24	rs12144621	CD247	0.036
T1D	1q24	rs863455	CD247	0.025
T1D	1q24	rs704852	CD247	0.029
T1D	6p21	rs9270986	HLA-DRB1	1.8x10^-6^
T1D	11p15	rs1004446	INS	0.011
T1D	11p15	rs6356	INS	0.020
T1D	11p15	rs7111341	INS	0.021
AITD	1q24	rs17534481	CD247	0.018
AITD	1q24	rs12095738	CD247	0.022
AITD	2q24	rs1990760	IFIH1	0.032
AITD	2q33	rs3087243	CTLA4	0.039
AITD	11p15	rs6356	INS	0.014
AITD	12q24	rs17696736	C12orf30	0.032

An association (*p*-value < 0.05) was found for 19 of the 79 SNPs on the GoldenGate Custom Panel with T1D or AITD using the family based association test (FBAT) (http://www.hsph.harvard.edu/fbat/default.html). *P*-values were not corrected for multiple testing

## Discussion

A GWLS and an association study was performed to replicate known T1D/AITD susceptibility loci/variations and to screen for novel loci in a multiplex family panel, which included family members affected by T1D and/or AITD, from northern Sweden. The AITD patient material in this study included both Graves’ disease (GD) and Hashimoto’s thyroid (HT) disease patients. Due to the aim of this study we did not attempted to analyze HT and GD as separate groups limiting the analysis to signals common to the two main groups included in the AITD group.

The initial linkage analysis revealed a LOD score > 1.5 in three regions on chromosomes 1q23, 4q25 and 6p21. The one-lod-drop region on chromosome 4 (4q23-4q27) harbors 264 genes (http://www.ncbi.nlm.nih.gov/projects/mapview) including the previously published susceptibility genes *IL2*/*IL21* [23, 38]. The one- lod-drop region on chromosome 6 (6p24-6p12) contains 1247 genes including the *HLA* genes and the one-lod-drop region on chromosome 1 (1q13-1q25) harbors 1378 genes including one very interesting novel candidate gene, *CD247*.

Next we performed an association analysis to replicate previously published associations as well as to elucidate if there was an association between variants in *CD247* and T1D/AITD. The follow-up association analysis confirmed associations with T1D for variants in *PTPN22*, *CD247, HLA* and *INS* and associations with AITD for variants in *CD247, IFIH1, CTLA4, INS* and *C12orf30* with nominal *p*-values < 0.05. *P*-values were not corrected for multiple testing. As expected, the most robust evidence of association was found for SNPs at the *HLA-DRB1* locus on chromosome 6, with rs9270986 displaying the lowest p-value (*p* = 1.8x10^-6^). After this association, the most robust evidence in favor of association was unexpectedly observed for rs2988276 in *CD247* on chromosome 1 (*p* = 3.8 × 10^-5^).

Variants in *PTPN22*, *HLA-DRB1*, *INS, IFIH1, CTLA4* and *C12orf30* have previously been associated with T1D [21, 23, 24, 33] and variants in most of the above genes have been associated to AITD [4, 14, 34, 39–42] and several other disorders [43–48]. No previous study has shown association between T1D/AITD and *CD247*, but variants in the gene have been associated with systemic lupus erythematosus (SLE) [49–52], rheumatoid arthritis (RA) [53] and systemic sclerosis [54].

*CD247* codes for CD3ζ, which is a component of the TCR/CD3 signaling complex on T cells, and tyrosine phosphorylation of CD3ζ is one of the first events occurring after TCR engagement [27, 28]. The CD3ζ subunit functions as an amplifier of TCR signaling and is also essential for efficient surface expression of the TCR/CD3 complex [55, 56]. Upon TCR engagement, CD8/CD4 delivers Lck to the CD3 complex, which leads to phosphorylation of ITAMs on CD3ζ. Phosphorylated CD3ζ acts as a docking site for Zap-70, which in turn, leads to the phosphorylation of Zap-70. Phosphorylated Zap-70 phosphorylates LAT, leading to downstream signaling (reviewed in [57]). ITAM-mediated signal amplification has been implicated as an important factor in the selection of the mature T-cell repertoire. Positive and negative selection in the thymus is impaired in CD3ζ-deficient mice reconstituted with transgenes encoding CD3ζ with mutated ITAMs. The requirement for ITAM-mediated signal amplification is most evident in the positive selection of T cells showing weak TCR-ligand interactions (reviewed in [58]). Holst et al. demonstrated that mice with reduced ITAMs in the TCR/CD3 complex develop a multi-organ autoimmune disease caused by a breakdown in central tolerance [59].

We have previously identified *Cd247* as a candidate susceptibility gene in murine T1D [60, 61] and we found that the NOD allele of *Cd247* confers impaired T cell activation, resulting in altered cytokine expression patterns, reduced proliferation and deficient CTLA-4 expression compared to the C57BL/6 (B6) allele of *Cd247* [60]. Altered expression and function of CD3ζ has also been implicated in autoimmune disorders, such as SLE [49, 62] and RA [63]. We did not find any association between T1D/AITD and variants in *IL2/IL21* despite the linkage peak observed in the 4q27 region. One possible explanation for this is that the linkage to T1D/AITD found in the region is caused by other genes or variants than the ones used in our association study.

One limitation of the current study is the relative modest size of the family-based materials employed. Genome-wide associations can be due to spurious causes, especially heterogeneity/population stratification.

## Conclusion

The fact that we have found association to T1D/AITD for five (*PTPN22, IFIH1, CTLA4, INS* and *C12orf30*) previously reported non-HLA genes as well as a novel susceptibility gene, *CD247*, supports the role for family-based studies in the search for genes involved in complex diseases. The results underpin the notion that the population of northern Sweden is well suited to the detection of genes involved in complex diseases. The use of our more restricted patient material, compared to other materials used in published GWA studies, enables the discovery of disease associated genes in a more cost effective manner and shows that our population is capable of detecting general susceptibility genes. The access to a genotyped family material with both affected and non-affected family members will be very important for future functional studies of associated gene variants.

## Additional file

Additional file 1: Table S1. Clinical characteristics of the study material. **Table S2.** HLA-DQB1 genotypes in the study material. **Table S3.** SNPs included in the GoldenGate Custom Panel. (PDF 338 kb)

## Abbreviations

AITD: Autoimmune thyroid disease
cM: centimorgan
GD: Graves’ disease
GWA: Genome-wide association
GWLS: Genome-wide linkage scan
HT: Hashimotos’ thyroiditis
ITAM: Immunoreceptor tyrosine-based activation motifs
LOD: Logarithm of odds
NOD: Non-obese diabetic
RA: Rheumatoid arthritis
SLE: Systemic lupus erythematosus
SNP: Single nucleotide polymorphism
T1D: Type 1 diabetes
TCR: T cell receptor
