# Supplementary material for: Association of CD247 (CD3ζ) gene polymorphisms with T1D and AITD in the population of northern Sweden
Source: BMC Med Genet. 2016 Oct 4;17:70. doi: 10.1186/s12881-016-0333-z (PMC5050583; doi:10.1186/s12881-016-0333-z)
Supplement: Additional file 1: Table S1. — Clinical characteristics of the study material. Table S2. HLA-DQB1 genotypes in the study material. Table S3. SNPs included in the GoldenGate Custom Panel. (PDF 338 kb) [file 12881_2016_333_MOESM1_ESM.pdf]

Supplementary table 1. Clinical characteristics of the study material.

	T1D		AITD		Both		Healthy	
	Female	Male	Female	Male	Female	Male	Female	Male
Gender *n* (%)	57 (46.3)	66 (53.7)	90 (81.8)	20 (18.2)	15 (75)	5 (25)	99 (48.1)	107 (51.9)
Median age at onset (years)	11	13.5	40.5	47	13	14		
Median duration of disease at sampling (years)	18	14	11	8	29	20		

Supplementary Table 1 – Clinical characteristics of the study material

Gender is reported as number (n) and percentage (%). Median age at onset and median duration of disease at sampling is reported as years.

Supplementary table 2. HLA-DQB1 genotypes in the study material.

	HLA-DQB1							
	*0302/*0302	*0302/*X	*0302/*0201	*0201/*0201	*0201/*X	*0302/*0602-04	*0201/*0602-04	Other
T1D (%)	1.9	33.3	37.0	0.0	7.4	7.4	3.7	9.3
AITD (%)	2.2	13.0	6.5	0.0	8.7	17.4	13.0	39.1
Both (%)	0.0	21.4	28.6	0.0	21.4	7.1	7.1	14.3
Healthy (%)	0.0	17.5	10.5	0.0	17.5	19.3	8.8	26.3

Supplementary Table 2 – HLA-DQB1 genotypes in the study material

HLA-DQB1 genotypes were analyzed in 172 of the individuals included in the study (55 with T1D, 46 with AITD, 14 with both disorders and 57 healthy individuals). X = any HLA-DQB1 allele besides *0302, *0201 or *0602-04. Other = any HLA-DQB1 haplotype besides *0302/*0302, *0302/*X, *0302/*0201, *0201/*0201, *0201/*X, *0302/*0602-04 or *0201/*0602-04.

Supplementary table 3. SNPs included in the GoldenGate Custom Panel.

SNP	Chr	Gene	Success rate %	p-value Model 1	p-value Model 3
rs672797	1p31	LPHN2	99	0.690	0.672
rs6679677	1p13	PTPN22	99	$4.5x10^{-4}$	0.112
rs2476601	1p13	PTPN22	99	$4.5x10^{-4}$	0.112
rs1052230	1q24	CD247	99	0.257	-
rs6668182	1q24	CD247	99	0.007	-
rs3108156	1q24	CD247	99	0.102	-
rs2995082	1q24	CD247	99	0.661	0.709
rs864537	1q24	CD247	99	0.143	0.778
rs7523907	1q24	CD247	99	0.761	0.516
rs2995089	1q24	CD247	99	0.401	0.707
rs2988276	1q24	CD247	99	$3.8x10^{-5}$	0.258
rs1723015	1q24	CD247	99	0.118	0.781
rs7523351	1q24	CD247	99	0.012	-
rs10918695	1q24	CD247	99	0.006	0.607
rs2995093	1q24	CD247	99	0.533	0.100
rs12128094	1q24	CD247	99	0.166	0.953
rs1214609	1q24	CD247	99	0.120	0.595
rs4657662	1q24	CD247	99	0.885	0.444
rs12737372	1q24	CD247	99	0.942	0.953
rs2949656	1q24	CD247	99	0.898	0.339
rs16859085	1q24	CD247	99	0.756	0.462
rs12144621	1q24	CD247	98	0.036	0.952
rs1214603	1q24	CD247	99	0.528	0.813
rs858553	1q24	CD247	99	0.570	0.711
rs863455	1q24	CD247	99	0.025	0.947
rs858547	1q24	CD247	97	0.457	0.267

rs858545	1q24	CD247	99	0.577	0.464
rs704848	1q24	CD247	98	0.469	0.894
rs704852	1q24	CD247	99	0.029	0.155
rs10918706	1q24	CD247	99	0.880	0.128
rs858543	1q24	CD247	99	0.489	0.947
rs704853	1q24	CD247	99	0.244	-
rs17534481	1q24	CD247	99	0.608	0.018
rs1737506	1q24	CD247	99	0.265	0.148
rs6686571	1q24	CD247	99	0.321	0.573
rs1799704	1q24	CD247	99	0.533	0.301
rs2982484	1q24	CD247	99	0.416	0.959
rs858550	1q24	CD247	99	0.346	0.117
rs12095738	1q24	CD247	99	0.717	0.022
rs2982481	1q24	CD247	99	0.346	0.136
rs1990760	2q24	IFIH1	99	0.399	0.032
rs3087243	2q33	CTLA4	99	0.928	0.039
rs6534347	4q26-27	IL-2	97	0.813	0.837
rs17388568	4q26-27	IL-2	98	0.565	0.961
rs17454584	4q26-27	Tenr-IL2-IL21	99	0.822	0.904
rs3136534	4q26-27	IL-2	99	0.883	0.652
rs4833248	4q26-27	Tenr-IL2-IL21	99	0.983	0.913
rs7667439	4q26-27	Tenr-IL2-IL21	99	0.655	0.983
rs13101493	4q26-27	Tenr-IL2-IL21	99	0.535	0.611
rs6822844	4q26-27	Tenr-IL2-IL21	99	0.369	0.334
rs7670387	4q26-27	Tenr-IL2-IL21	99	0.668	0.898
rs13143866	4q26-27	Tenr-IL2-IL21	99	0.461	0.503
rs12508721	4q26-27	Tenr-IL2-IL21	99	0.902	0.905
rs6819371	4q26-27	Tenr-IL2-IL21	99	0.968	0.724
rs6897932	5p13	IL7R	99	0.140	0.933
rs9270986	6p21	HLA-DRB1	97	$1.8x10^{-6}$	0.305

rs213950	7q31	CFTR	99	0.583	0.378
rs706778	10p15	IL2Ra	99	0.239	0.709
rs11597367	10p15	IL2Ra	99	0.122	0.380
rs41295061	10p15	IL2Ra	99	0.431	-
rs11594656	10p15	IL2Ra	99	0.122	0.380
rs12251307	10p15	IL2Ra	99	0.074	0.262
rs3741208	11p15	INS	79	0.810	0.367
rs1004446	11p15	INS	99	0.011	0.795
rs6356	11p15	INS	99	0.020	0.014
rs10770141	11p15	INS	99	0.119	0.299
rs10743152	11p15	INS	99	0.086	0.299
rs7111341	11p15	INS	99	0.021	0.334
rs11171739	12q13	ERBB3	99	0.278	0.550
rs2292239	12q13	ERBB3	99	0.430	0.858
rs3184504	12q24	SH2B3	99	0.833	0.137
rs17696736	12q24	C12orf30	99	0.896	0.032
rs725613	16p13	KIAA0350	94	0.414	0.850
rs12708716	16p13	KIAA0350	99	0.390	0.903
rs2903692	16p13	KIAA0350	99	0.539	0.513
rs17673553	16p13	KIAA0350	99	0.073	0.545
rs2542151	18p11	PTPN2	99	0.438	0.595
rs1893217	18p11	PTPN2	99	0.438	0.595
rs763361	18q22	CD226	99	0.843	0.398

Supplementary Table 3 - SNPs included in the GoldenGate Custom Panel.

Family based association test (FBAT) (http://www.hsph.harvard.edu/fbat/default.html) was used to test association of the 79 SNPs on the GoldenGate Custom Panel. In disease model 1, individuals with T1D were set as affected. In disease model 3, individuals with AITD were set as affected. A p-value<0.05 indicates association. P-values were not corrected for multiple testing. Uninformative SNPs in the model are denoted –.
